# Microbial Musings – August 2021

**DOI:** 10.1099/mic.0.001107

**Published:** 2021-09-22

**Authors:** Gavin H. Thomas

**Affiliations:** ^1^​ Department of Biology, University of York, YO10 5YW, UK

In the month that many of us in the UK and Europe take some well-earned time off, I’ve been reading a great new book, *Vaxxers*, which tells the story of the Oxford–AstraZeneca vaccine from the personal perspective of two of the leading protagonists, Catherine Green (@CathGreenLab) and Sarah Gilbert, both from the University of Oxford, UK [1]. As well as highlighting the work of two excellent life scientists, the people-based narrative of the events of 2020 tells the gripping story of how the Oxford researchers were able to rapidly mobilize, raise funds, design and manufacture the vaccine, and then partner with Big Pharma to deliver the first vaccine to be used in the UK during the pandemic. One hopes that many of the public do read this book, as it most comprehensively debunks any dogma of scientists working as individuals in ivory towers, with an exposition of the realities of an extraordinarily well-prepared group of researchers trying to fast track a new vaccine under incredible pressure. It even features young microbiologist Eliza Grenato (@Prokaryota), who was one of the first of two people to receive the vaccine at the start of clinical trials and who had to soon after deny a rumour that she had passed away – there are some good chapters on misinformation and some of the lessons the authors learnt about this, too, which are well worth a read for all of us. It was very gratifying to see Sarah Gilbert, now Dame Sarah Gilbert, receive a standing ovation when she attended Wimbledon 2021 – clearly the vast majority of people are incredibly grateful for the efforts of scientists in developing this and other vaccines so quickly in what has been a remarkably successful period of scientific development.

This period will, I suspect, give historians of science plenty of material to work with and is an example of where rapid application of scientific knowledge has impacted immediately on public health. As you will know if you have been reading these articles over the last year, we are gearing up for our 75th Anniversary in 2022 and indeed have our own historian of science, Peter Collins, working on the archive of the Microbiology Society to find material relevant to *Microbiology* and its history. We are developing a series of themes to highlight microbiological stories that start around the time of the foundation of the journal in 1947. In this period, as the UK emerged from the end of the Second World War, there were also many challenges that microbiological research was trying to address, including the early use and manufacture of antibiotics, understanding bacterial pathogenesis and the working out of many of the biochemical pathways that underpin life. Some of these themes will link to our exciting two-day 75th Symposium that will take place at Annual Conference at Belfast in 2022. An exciting time for us to look back at the impact of *Microbiology* and microbiology over the last three-quarters of a century and look forward to its future.

We start the science this month with two papers thinking about how bacteria access sugars in their environment. The first is a paper that just missed out on being included in last month’s *Musings* as it sneaked into the July issue after I’d written my piece. This is an article from the group of new *Microbiology* editor Nicola Holden (@NicolaJHolden) from the James Hutton Institute in Dundee (@HuttonCMS) [2], led by Louise Crozier (@LouiseECrozier) and in collaboration with Robert Jackson (@RobWJackson) from the University of Reading. The paper concerns the foodborne pathogen *Escherichia coli* O157:H7 and how it can survive on plants, which they use as secondary hosts, allowing transmission through the food chain. Plant cell walls, a bit like human gut mucin, provide various sugars that could be used as food for *E. coli *, but normal gut commensal strains of *E. coli* are known to be remarkably bad at exploiting complex polysaccharides and generally only access monosaccharides and limited disaccharides, relying on other microbes in their niche to do the leg work in liberating these from longer polymers. In my lab we discovered that *E. coli* even makes dedicated transporters for very rare forms of monosaccharides, but still leaves the degradation of more complex glycans to others [3, 4]. One monosaccharide that *E. coli* K-12 uses efficiently is l-arabinose (l-Ara), a sugar that is mainly derived from plant biomass in arabino-xylan and pectin components of the plant cell wall. In this work the group aim to study if the O157:H7 strain of *E. coli *, which can colonize plants efficiently, is any better at accessing l-Ara tied up in plant polysaccharides. They find all the usual genes known from K-12 strains to be present and using *gfp* fusions demonstrate that their expression is induced by l-Ara specifically. A strain lacking the main catabolic genes is still able to colonize plants normally, but this is perhaps not a surprise given that l-Ara is not the only source of carbon available. Growth in liquid culture on a variety of plant leaf extracts did not strongly induce expression of the *gfp* fusion, suggesting that limited free l-Ara is present in these extracts, although when examined *in planta*, where other natural leaf microbiome components were present, induction of the genes could be seen in individual cells, suggesting that they can detect free l-Ara perhaps released by the action of other components of the microbiome. Finally, they find that l-Aara-containing oligosaccharides derived from the pectin component arabinan were able to induce expression of the *ara* genes and could also support some growth in minimal medium as the sole source of carbon, suggesting that there could be some additional transport and degradation capabilities compared to K-12 strains, but overall that O157:H7 has not particularly adapted its l-Ara metabolism to help it during transmission on its secondary host.

In the second paper in this area we look at the metabolism of another disaccharide derived from plant cell walls, namely cellobiose, which contains two glucose molecules that would have been produced from enzymatic cleavage of cellulose. While environmental clostridia are known to be very good at eating cellulose, and in fact the cellulosome was discovered in a *Clostridium* sp. [5], this study from the group of Revathi Govind and colleagues at Kansas State University, USA (@kstatebio) looks at how the human pathogen *Clostridiodes difficile* uses cellobiose and how it is taken up into the cell [6]. They study the most likely candidate for this process, a phosphotransferase (PTS)-type transporter that concomitantly transports and phosphorylates the sugar, and which are well known to be the primary type of transporter used for sugar uptake in clostridia from the work of Wilf Mitchell from Herriot Watt University, UK [7–9]. The authors disrupt the genes for the candidate transporter and indeed find that growth on cellobiose as the sole carbon source is lost. They then consider how the expression of the transporter is regulated and focus on a novel transcriptional repressor protein, CelR, that they find binds upstream of the promoter region of the genes encoding the transporter (*cel* genes). Interestingly, as well as the normal growth phenotype, they also find that cellobiose transporter mutant strain has reduced sporulation efficiency, and that when they use the mutant in an *in vivo* hamster challenge experiment they see a significant reduction in pathogenicity of the strain in both primary and recurrent infection models. As we saw in the l-ara study just mentioned, and in many other studies on sugar metabolism in bacteria, removal of a single system often has little *in vivo* phenotype due to the redundancy and complexity of sugars available to the bacterium, and so a specific reduction in both sporulation and pathogenicity suggests a more important function for cellobiose, perhaps as an environmental signal as well as just a simple nutrient.

We switch to industrial biotechnology for our next paper, which is about understanding factors that lead to lipogenesis in the fungus *Mortierella alpina* [10]. This is an oleaginous microbe, meaning that under certain growth conditions it can accumulate lipids to very high levels and this species can remarkably reach nearly 50 % of its dry weight as lipids. The authors from the group of Wei Chen and Jianxin Zhao, from Jiangsu University, PR China, had already looked at some of the main routes of metabolism that produce NADPH, a key metabolite required for lipid production, and found that pentose phosphate pathways enzymes were important [11]. Here they look at a metabolic pathway not often found in fungi, which breaks down phenylalanine to TCA cycle intermediates, and find that it does appear to contribute to the high levels of lipids being accumulated. It would appear that the flux to the TCA intermediates likely provides substrates for the malic enzyme, a known provider of NADPH in oleaginous microbes [12, 13] and removal of this would lead to the observed reduced ability to accumulate lipids. This is a nice example of where less obvious metabolic pathways make a contribution to an overall metabolic phenotype relevant for biotechnology.

The final paper to highlight this month is a review article, also with an applied nature, about the delivery of oral probiotics [14]. Our oral hygiene is something we all intervene with daily when we clean our teeth and products to help us improve oral health abound. This article from the group of Siok-Koon Yeo at Taylor’s University, Malaysia (@Taylors_Uni) introduces us first to the oral microbiome and its role in disease and then collates existing efforts to use probiotics to improve oral health. This mainly features strains of *Streptoccocus salivarius* and various *Lactobacillus* sp., which are normal components of the oral microbiome not linked to disease and with the ability to produce antimicrobials such as bacteriocins [15]. How the bacterial components survive in these preparations, stick around in the mouth and actively inhibit oral pathogens is covered in the article. Finally the mechanisms of delivery are reviewed, with bacteria being added to lozenges, capsules, food, mouthwashes and gels to get the microbes in the right place. That’s all for this month and we should have a bumper crop of papers for you in next month’s article.

## References

[1] Gilbert S, Green C (2021). Vaxxers: The Inside Story of the Oxford Astrazeneca Vaccine and the Race Against the Virus.

[2] Crozier L, Marshall J, Holmes A, Wright KM, Rossez Y (2021). The role of l-arabinose metabolism for Escherichia coli O157:H7 in edible plants. Microbiology.

[3] Horler RSP, Müller A, Williamson DC, Potts JR, Wilson KS (2009). Furanose-specific sugar transport: characterization of a bacterial galactofuranose-binding protein. J Biol Chem.

[4] Maqbool A, Horler RSP, Muller A, Wilkinson AJ, Wilson KS (2015). The substrate-binding protein in bacterial ABC transporters: Dissecting roles in the evolution of substrate specificity. Biochem Soc Trans.

[5] Fontes CMGA, Gilbert HJ (2010). Cellulosomes: Highly efficient nanomachines designed to deconstruct plant cell wall complex carbohydrates. Annu Rev Biochem.

[6] Hasan MK, Dhungel BA, Govind R (2021). Characterization of an operon required for growth on cellobiose in clostridioides difficile. Microbiology.

[7] Mitchell WJ, Albasheri KA, Yazdanian M (1995). Factors affecting utilization of carbohydrates by clostridia. FEMS Microbiol Rev.

[8] Mitchell WJ, Roohi MS, Mosely MJ, Booth IR (1987). Regulation of carbohydrate utilization in *Clostridium pasteurianum*. Microbiology.

[9] Mitchell WJ, Booth IR (1984). Characterization of the *Clostridium pasteurianum* phosphotransferase system. Microbiology.

[10] Wang H, Wang C, Yuan W, Chen H, Lu W (2021). The role of phenylalanine hydroxylase in lipogenesis in the oleaginous fungus *Mortierella alpina*. Microbiology.

[11] Chen H, Hao G, Wang L, Wang H, Gu Z (2015). Identification of a critical determinant that enables efficient fatty acid synthesis in oleaginous fungi. Sci Reports.

[12] Wynn JP, Ratledge C (1997). Malic enzyme is a major source of nadph for lipid accumulation by *Aspergillus nidulans*. Microbiology (Reading).

[13] Ratledge C (2014). The role of malic enzyme as the provider of nadph in oleaginous microorganisms: A reappraisal and unsolved problems. Biotechnol Lett.

[14] How Y-H, Yeo S-K (2021). Oral probiotic and its delivery carriers to improve oral health: A review. Microbiology.

[15] López-López A, Camelo-Castillo A, Ferrer MD, Simon-Soro Á, Mira A (2017). Health-associated niche inhabitants as oral probiotics: The case of streptococcus dentisani. Front Microbiol.

